# The SARS coronavirus E protein interacts with the PALS1 and alters tight junction formation and epithelial morphogenesis

**Published:** 2011-01-10

**Authors:** Kim-Tat Teoh, Yu-Lam Siu, Wing-Lim Chan, Marc A Schlüter, Chia-Jen Liu, J S Malik Peiris, Roberto Bruzzone, Benjamin Margolis, Béatrice Nal

**Affiliations:** 1 HKU-Pasteur Research Centre, Hong Kong, Hong Kong SAR; 2 Medizinische Klinik D, Universitätsklinikum Münster, D-48149 Münster, Germany; 3 Department of Biological Chemistry, University of Michigan Medical School, Ann Arbor, MI 48109, USA; 4 Department of Microbiology, Li Ka Shing Faculty of Medicine, The University of Hong Kong, Hong Kong SAR; 5 Department of Internal Medicine, University of Michigan Medical School, Ann Arbor, MI 48109, USA; 6 Department of Anatomy, Li Ka Shing Faculty of Medicine, The University of Hong Kong, Hong Kong SAR

## 

Intercellular tight junctions define epithelial apicobasal polarity and form a physical fence which protects underlying tissues from pathogen invasions. PALS1, a tight junction-associated protein, is a member of the CRUMBS3-PALS1-PATJ polarity complex, which is crucial for the establishment and maintenance of epithelial polarity in mammals. Here we report that the carboxy-terminal domain of the SARS-CoV E small envelope protein (E) binds to human PALS1. Using co-immunoprecipitation and pull-down assays, we show that E interacts with PALS1 in mammalian cells and further demonstrate that the last four carboxy-terminal amino-acids of E form a novel PDZ-binding motif that binds to PALS1 PDZ domain. PALS1 redistributes to the virion assembly site, where E is enriched, in SARS-CoV-infected Vero E6 cells. Ectopic expression of E in MDCKII epithelial cells significantly alters cellular polarity and induces formation of cysts with multiple lumens. We show that E expression delays formation of tight junctions and affects the subcelullar distribution of the apical and tight junction markers GP135 and ZO-1, respectively. We speculate that hijacking of PALS1 by SARS-CoV E plays a determinant role in the disruption of the lung epithelium in SARS patients.

